# Supplementary material for: A hypothalamic-thalamostriatal circuit that controls approach-avoidance conflict in rats
Source: Nat Commun. 2021 May 4;12:2517. doi: 10.1038/s41467-021-22730-y (PMC8097010; doi:10.1038/s41467-021-22730-y)
Supplement: Supplementary file 1 — Supplementary Information [file 41467_2021_22730_MOESM1_ESM.pdf]

Supplementary 1

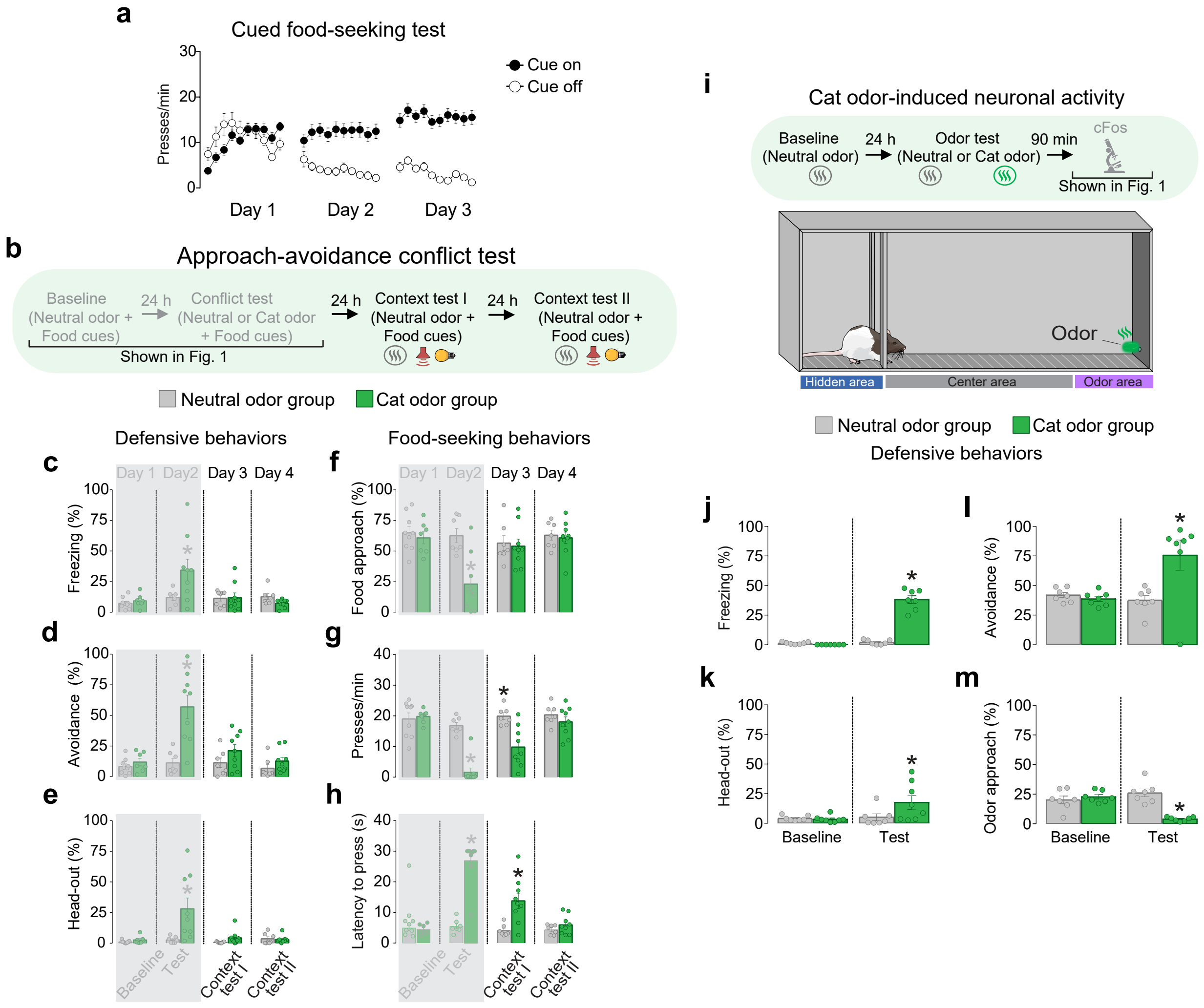

Supplementary Figure 1 (Related to Figure 1). Rats exposed to the conflict test showed long-lasting suppression in food-seeking responses in the same context.

(a) Rats previously trained to press a lever for sucrose in a variable interval schedule of reinforcement (1 to 60 s) were exposed to a cued lever pressing training for three days. After 3 days of training, lever press rates increased during the cue-on period (black dots, blocks of 2 trials) when compared to the cue-off period (white dots). **(b)** Timeline and schematic of the complete approach-avoidance conflict test. After the baseline and conflict test (results covered by gray shadow, also shown in **Fig.1**), rats went through two consecutive days of test in the same context in the presence of neural odor and food cues (context test I and context test II). **(c-h)** Rats in the cat odor group (green bars, *n* = 9) showed a reduction in (g) lever presses ($F_{(3, 42)}$ = 11.71, P < 0.001) with an increase in the (h) latency to press the lever ($F_{(3, 42)}$ = 25.67, P < 0.001) during the context test I. No changes were observed in (c) freezing, (d) avoidance, (e) head-out, and (f) food approach (all P's > 0.099), when compared to neutral odor controls (gray bars, *n* = 7). **(i-m)** Naïve rats previously habituated in the odor chamber were exposed to either neutral odor or cat odor for 10 min, and then perfused 90 min later for immunohistochemical quantification of cFos expression (results shown in **Fig.1**). (i) Timeline of the cat odor-induced neuronal activity test. Cat odor exposure (green bars, *n* = 7) increased the percentage of time rats spent exhibiting (j) freezing ($F_{(1, 12)}$ = 126.6, P < 0.001), (k) avoidance ($F_{(1, 12)}$ = 11.45, P = 0.0054), and (l) head-out ($F_{(1, 12)}$ = 4.48, P = 0.0557 with Bonferroni planned comparison test p = 0.013) responses, and decreased (m) odor approach ($F_{(1, 12)}$ = 86.54, P < 0.001), when compared to neutral odor controls (gray bars, *n* = 7). Two-way repeated measures ANOVA followed by Bonferroni post hoc test. Data shown as mean ± SEM. *p < 0.05.

Supplementary 2

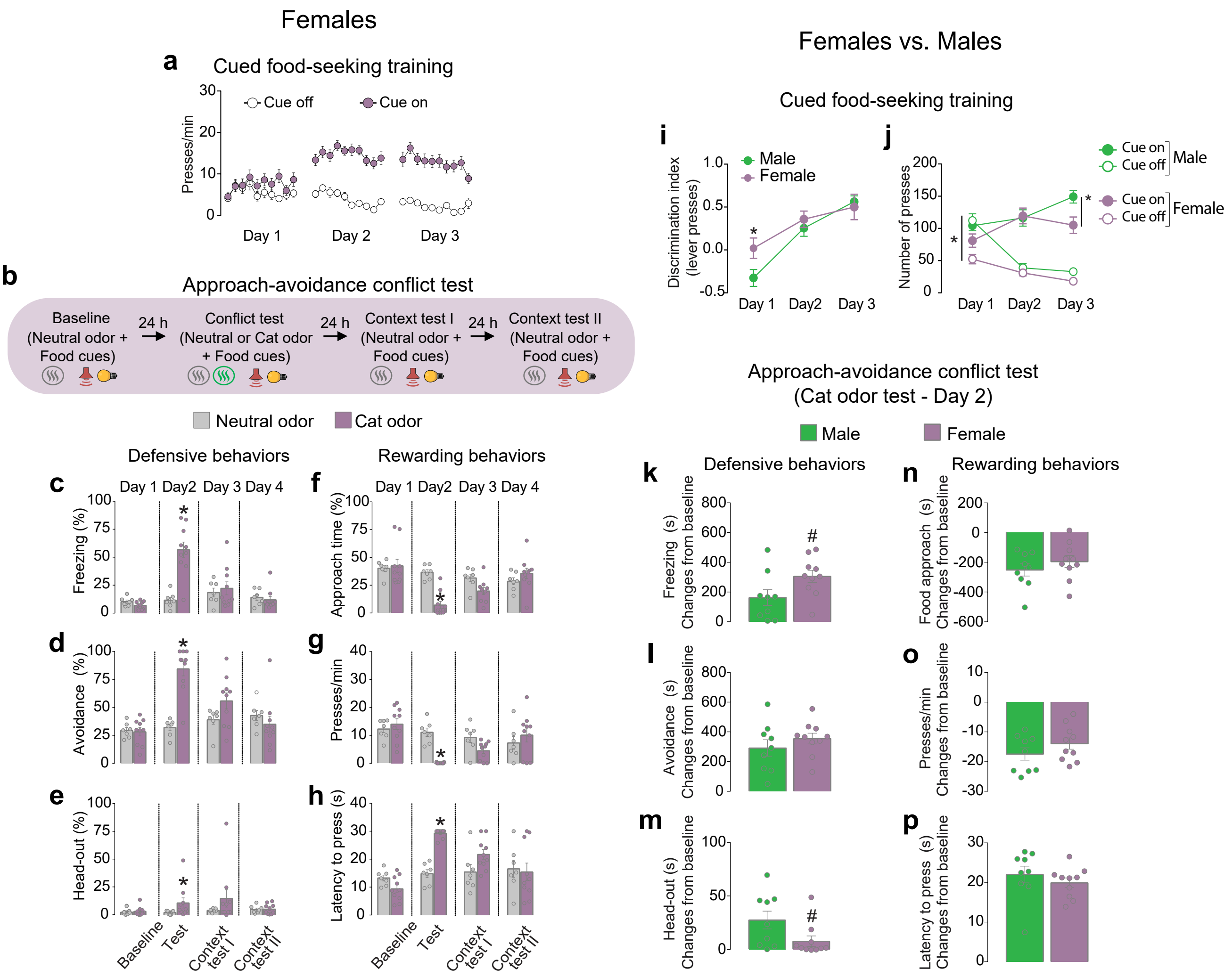

Supplementary Figure 2 (Related to Figure 1). Effects of sex differences on the approach-avoidance conflict test.

(a) Cued lever press training for female rats (n= 20). After 3 days of training, lever press rates increased during the cue-on period (lilac dots, blocks of 2 trials) when compared to the cue-off period (white dots). **(b)** Timeline and schematic of the complete approach-avoidance conflict test as described in Supplementary Fig 1b. **(c-h)** During the conflict test, female rats exposed to cat odor (lilac bars, *n* = 10) showed an increase in defensive behaviors characterized by an augment in the percentage of time exhibiting (c) freezing ($F_{(3, 42)}$ = 13.96, P < 0.001), (d) avoidance ($F_{(3, 42)}$ = 12.34, P < 0.001) and (e) head-out ($F_{(3, 42)}$ = 6.625, P < 0.001); and a decrease in food-seeking responses characterized by a reduction in the percentage of time (f) approaching the food area ($F_{(3, 42)}$ = 8.57, P < 0.001), a suppression in the number of (g) lever presses ($F_{(3, 42)}$ = 9.65, P < 0.001), and an increase in the (h) latency to press the lever ($F_{(3, 42)}$ = 10.28, P < 0.001) when compared to neutral odor controls (gray bars, *n*= 7; two-way repeated measures ANOVA followed by Bonferroni post hoc test). **(i)** Female rats (lilac solid circle, *n* = 20) showed a better discrimination index (*lever presses during cue-on minus lever presses during cue-off divided by total presses*) during day 1 of cued food-seeking training, when compared to male rats (green solid circle, *n* = 18; $F_{(2, 72)}$ = 1.585, P = 0.21, with Bonferroni planned comparison test p = 0.040). **(j)** Female rats pressed significantly less the lever during both cue-off trials on day 1 (lilac hollow circle) and cue-on trials on day 3 (lilac solid circle), when compared to male rats (green hollow circle and green solid circle, respectively; $F_{(6, 144)}$ = 14.6, P < 0.001). Two-way ANOVA followed by Bonferroni post hoc test. *p < 0.05. **(k-p)** Female and male rats showed similar changes in defensive behaviors and food-seeking behaviors during the conflict test compared to baseline. Female rats (lilac bars, *n* = 9) showed a trend to exhibit less (k) freezing (P = 0.058, *t* = 2.03) and (m) head-out responses (P = 0.063, *t* = 1.98), with no differences in (l) avoidance time (P = 0.584, *t* = 0.56), (n) food approach time (P = 0.335, *t* = 0.99), (0) lever presses (P= 0.227, *t* = 1.25), and (p) latency to press the lever (P = 0.389, *t* = 0.885), when compared to male rats (green bars, *n* = 9). Unpaired Student's t test. Data shown as mean ± SEM. *p < 0.05; $^{\#}$p between 0.05 and 0.099.

Supplementary 3

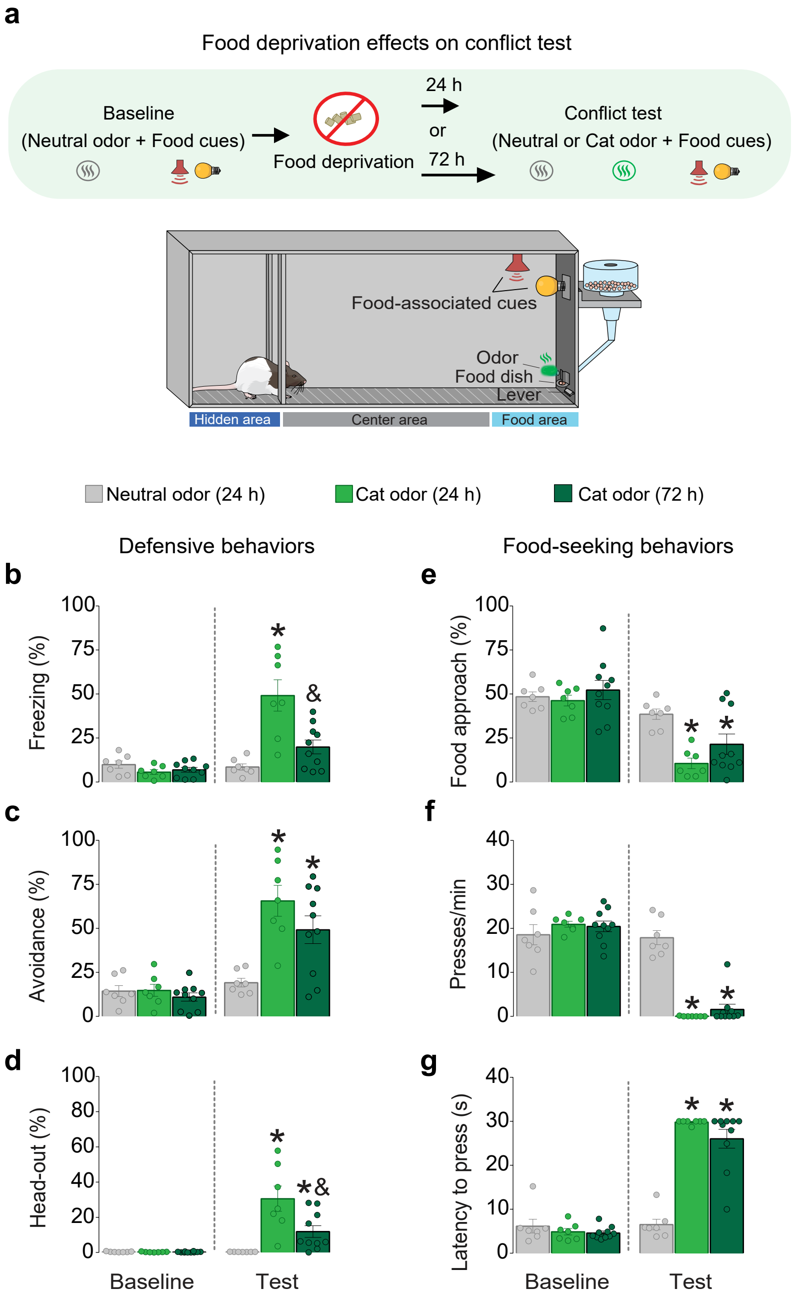

Supplementary Figure 3 (Related to Figure 1). Effects of different regimens of food deprivation on the approach-avoidance conflict test.

(a) Timeline and schematic of the food deprivation and approach-avoidance conflict test. After the baseline test, rats were exposed to either 24 h or 72 h of food deprivation in their home cages before being exposed to the conflict test. **(b-g)** Rats exposed to 24 h of food deprivation (light green bars, n = 7) showed an increase in defensive behaviors characterized by an augment in the percentage of time exhibiting freezing, avoidance, and head-out responses, and a decrease in food-seeking behaviors characterized by a reduction in the percentage of time approaching the food area, a suppression in the number of lever presses during food cues, and a prolonged latency to press the lever, when compared to neutral odor controls (gray bars, n = 7, all P's < 0.05). Rats exposed to 72 h of food deprivation (dark green bars, *n* = 10) showed a partial reduction in cat odor-induced defensive behaviors characterized by a decrease in (b) freezing ($F_{(2, 21)} = 15.5$, $P < 0.001$) and (d) head-out responses ($F_{(2, 21)} = 10.99$, $P < 0.001$) when compared to the cat odor group exposed to 24 h of food deprivation. No changes in (c) avoidance time or (e-g) food-seeking responses were observed between the cat odor groups exposed to 24 h or 72 h of food deprivation (all P's > 0.05), with both groups showing a reduction in (e) food approach ($F_{(2, 21)} = 7.37$, $P = 0.0037$), a suppression in (f) lever presses ($F_{(2, 21)} = 46.72$, $P < 0.001$), and an increase in the (c) avoidance time ($F_{(3, 42)} = 14.01$, $P<0.001$) and (g) latency to press the lever ($F_{(2, 21)} = 51.04$, $P < 0.001$), when compared to neutral odor controls. Two-way repeated measures ANOVA followed by Bonferroni post hoc test. Data shown as mean ± SEM. *p < 0.05 compared to 24 h neutral odor controls, $^{\&}$p < 0.05 compared to cat odor 24 h food deprivation group.

Supplementary 4

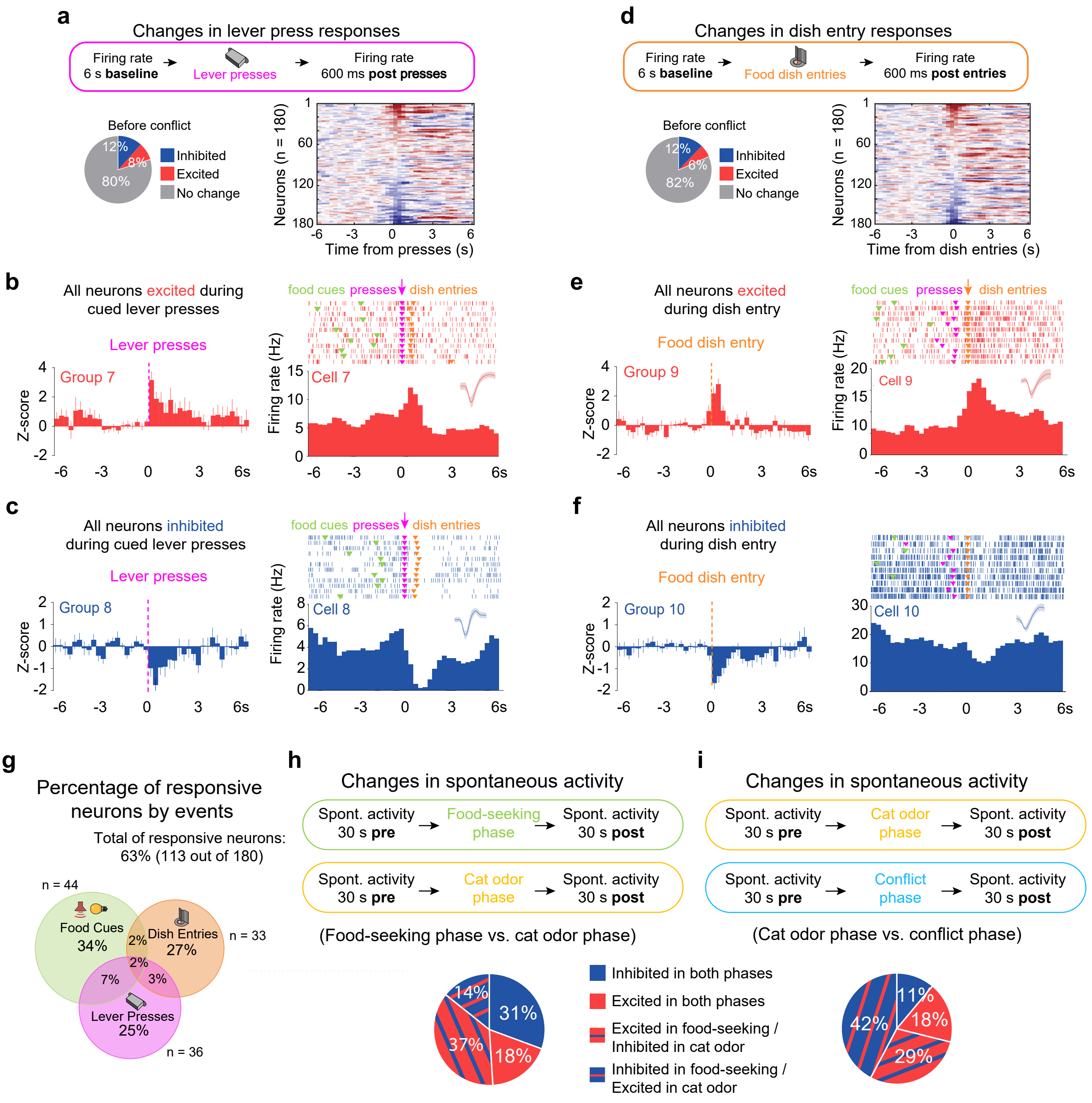

Supplementary Figure 4 (Related to Figure 2). Distinct subpopulations of aPVT neurons change their firing rate in response to food cues, dish entries, and lever presses.

(a) Top: Schematic of the recordings during cued lever presses-evoked responses. Bottom left, Pie charts showing the percentage of lever press responsive neurons before conflict. Bottom right, Heatmap showing the normalized firing rate of individual aPVT neurons time-locked for lever presses. **(b)** Left, Average PSTH of all aPVT neurons showing excitatory lever-press responses. Right, Raster plot and PSTHs of representative aPVT neuron showing excitatory lever-press responses. **(c)** Same as b, but for inhibitory lever-press responses. **(d)** Top: Schematic of the recordings during dish entry-evoked responses. Bottom left, Pie charts showing the percentage of dish entry responsive neurons before conflict. Bottom right, Heatmap showing the normalized firing rate of individual aPVT neurons time-locked for dish entry. **(e)** Left, Average PSTH of all aPVT neurons showing excitatory dish-entry responses. Right, Raster plot and PSTHs of representative aPVT neuron showing excitatory dish-entry responses. **(f)** Same as in e, but for inhibitory dish-entry responses. **(g)** Venn diagram showing the percentage of all responsive neurons (144 out of 180 neurons) by events. Most of the responsive neurons responded exclusively to one of the events. **(h)** Top: Schematic of the spontaneous activity recordings. Bottom: Pie chart showing the percentage of aPVT neurons that changed their baseline spontaneous activity (30 s pre vs. 30 s post) during the food-seeking phase vs. cat odor phase. **(i)** Same as in h, but comparing changes in spontaneous activity during the cat odor phase vs. conflict phase. n = 180 aPVT neurons from 19 rats.

Supplementary 5

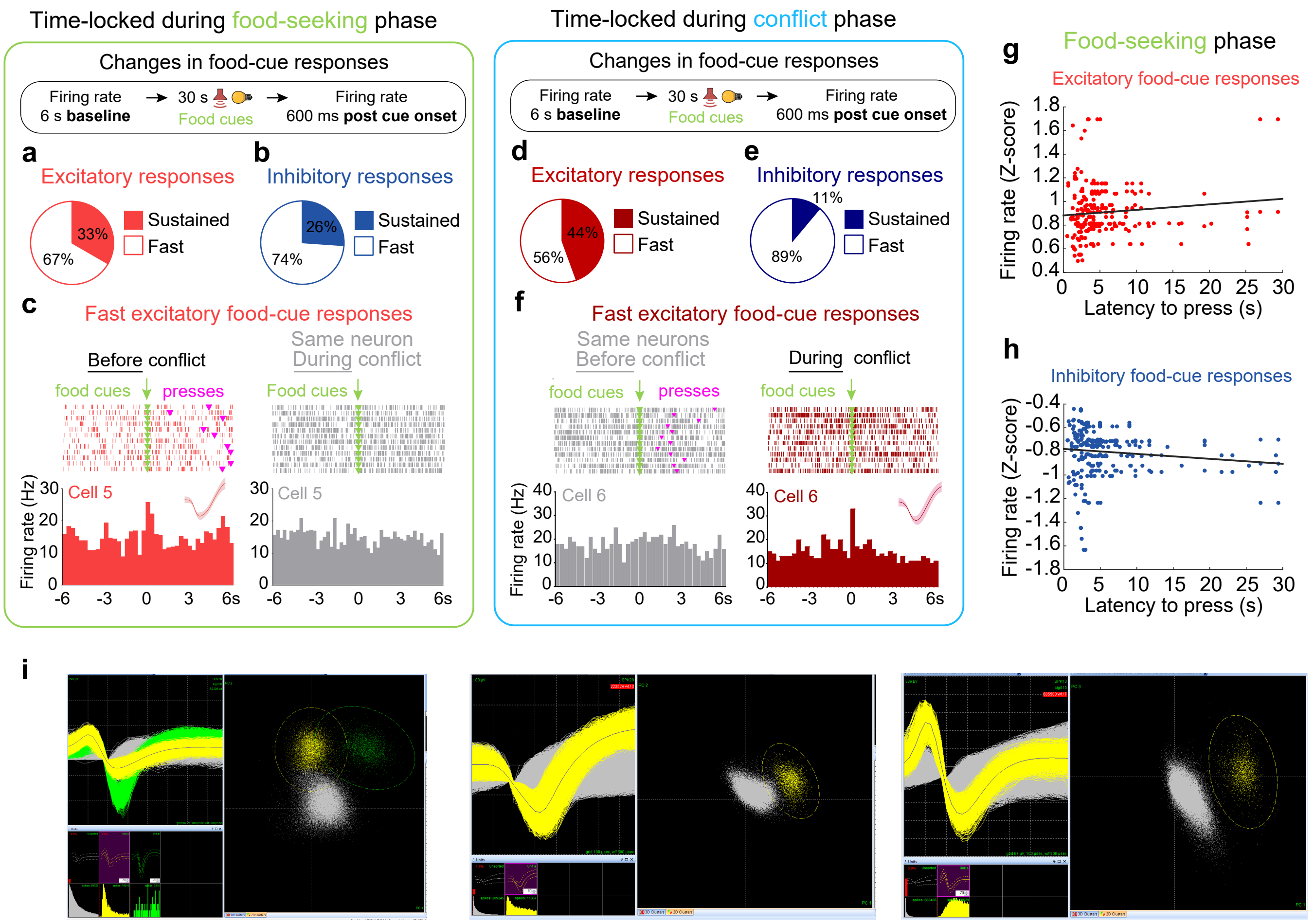

Supplementary Figure 5 (Related to Figure 2). Distribution of sustained and fast food-cue responses in aPVT neurons and their correlation with latency to press.

(a-b) Pie charts showing the percentage of aPVT neurons showing excitatory (a) or inhibitory (b) sustained food-cue responses (for longer than 1.2 seconds) or fast food-cue responses (for shorter than 1.2 seconds) time-locked during the food-seeking phase (Z-score > 2.58 for excitatory and < -1.96 for inhibitory responses, bins of 300 ms). **(c)** Raster plot and PSTH of representative aPVT neuron showing fast excitatory food-cue responses before conflict (red) and the same cell during conflict (gray). **(d-e)** Same as in a-b, but time-locked during the conflict phase. **(f)** Raster plot and PSTH of representative aPVT neuron showing fast excitatory food-cue responses during conflict (dark red) and the same cell before conflict (gray). **(g-h)** Scatter plot showing lack of correlation between normalized firing rate (Z-score) and latency to press (s) during the food-seeking phase. Analyses were divided into excitatory (g) or inhibitory (h) food-cue responses. Each data point denotes the averaged Z-scored response of one neuron until the animal pressed the lever (Y axis) vs. the respective latency to press during each food-cue presentation (X axis). The black line represents the linear regression of the data points, fitting the first-degree polynomial to the data (see Methods for details). **(i)** Raw data showing waveforms and principal component analyses in 3 representative channels. Sorted neurons are identified in yellow or green colors whereas unsorted spikes are shown in gray. The equation for the linear fitting in the excitatory case is y = 0.0047*x + 0.88 (r= 0.11, P = 0.09) and for inhibitory cases is y = - 0.0042*x - 0.78 (r= -0.12, P = . Pearson's correlation performed per animal and averaged: for excitatory responses - Average Pearson's r= 0.38 ± 0.08, P = 0.78 ± 0.04; for inhibitory responses - Average Pearson's r = 0.41 ± 0.09, P = 0.77 ± 0.04.

Supplementary 6

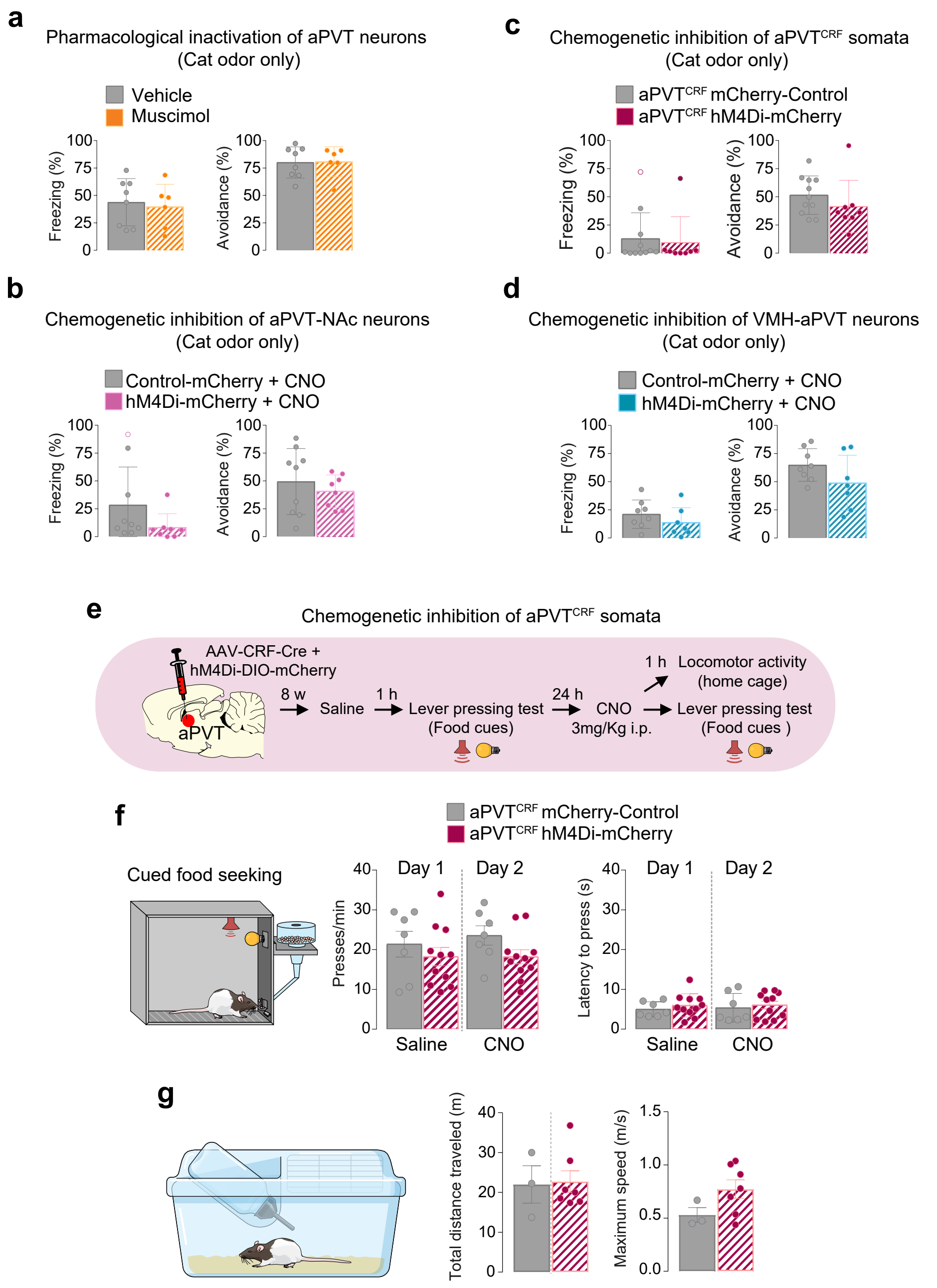

Supplementary Figure 6 (Related to Figure 3, 5, 9). Inactivation of aPVT neurons or aPVT inputs/outputs has no effect in the absence of conflict.

(a) Muscimol inactivation of aPVT neurons (orange stripped bars, n = 6) during cat odor exposure alone had no effect on the percentage of time rats spent exhibiting freezing (left, P = 0.72, *t* = 0.36) or avoidance responses (right, P = 0.93, *t* = 0.08), when compared to vehicle controls (gray bars, *n* = 8). **(b)** Chemogenetic inhibition of aPVT-NAc neurons (pink stripped bars, n = 8) during cat odor exposure alone had no effect on the percentage of time rats spent exhibiting freezing (left, P = 0.13, *t* = 1.57) or avoidance responses (right, P = 0.46, *t* = 0.75), when compared to mCherry controls (gray bars, *n* = 9). **(c)** Chemogenetic inhibition of $aPVT^{CRF}$ neurons (red wine stripped bars, n = 8) during cat odor exposure alone had no effect on the percentage of time rats spent exhibiting freezing (left, P = 0.75, *t* = 0.31) or avoidance responses (right, P = 0.28, *t* = 1.09), when compared to mCherry controls (gray bars, *n* =11). **(d)** Chemogenetic inhibition of VMH-aPVT projections (blue stripped bars, n = 7) during cat odor exposure alone had no effect on the percentage of time rats spent exhibiting freezing (left, P = 0.28, *t* = 1.11) or avoidance responses (right, P = 0.14, *t* = 1.55), when compared to mCherry controls (gray bars, *n* = 8). Results in **b-d** correspond to the first 10 min of the conflict session (Fig. 3h-n, Fig. 5a-g, and Fig. 9g-m respectively) in which rats were exposed to cat odor alone in the absence of food cues. All groups were microinjected with the iDREADDs ligand clozapine-N-oxide (CNO, 3 mg/Kg, i.p.) 1 hour before the test. Unpaired Student's t test. **(e)** Timeline of the lever pressing test during chemogenetic inhibition of $aPVT^{CRF}$ neurons. **(f)** Rats were placed into the cued food-seeking apparatus (left) and the number of lever presses and the latency to press in the presence of food cues were measured. Chemogenetic inhibition of $aPVT^{CRF}$ neurons (red wine striped bars, n = 11) had no effect on the number of lever presses (center, ($F_{(1, 16)}$ = 0.89, P = 0.35) or latency to press the lever (right, ($F_{(1, 16)}$ = 0.02, P = 0.86), when compared to mCherry controls (gray bars, n = 7). **(g)** Chemogenetic inhibition of $aPVT^{CRF}$ neurons (red wine striped bars, n = 7) had no effect on locomotor activity measured as total distance traveled and maximum speed in the home cage one hour after the CNO infusion, when compared to mCherry controls (gray bars, n = 3). Two-way repeated measures ANOVA. Data shown as mean ± SEM.

Supplementary 7

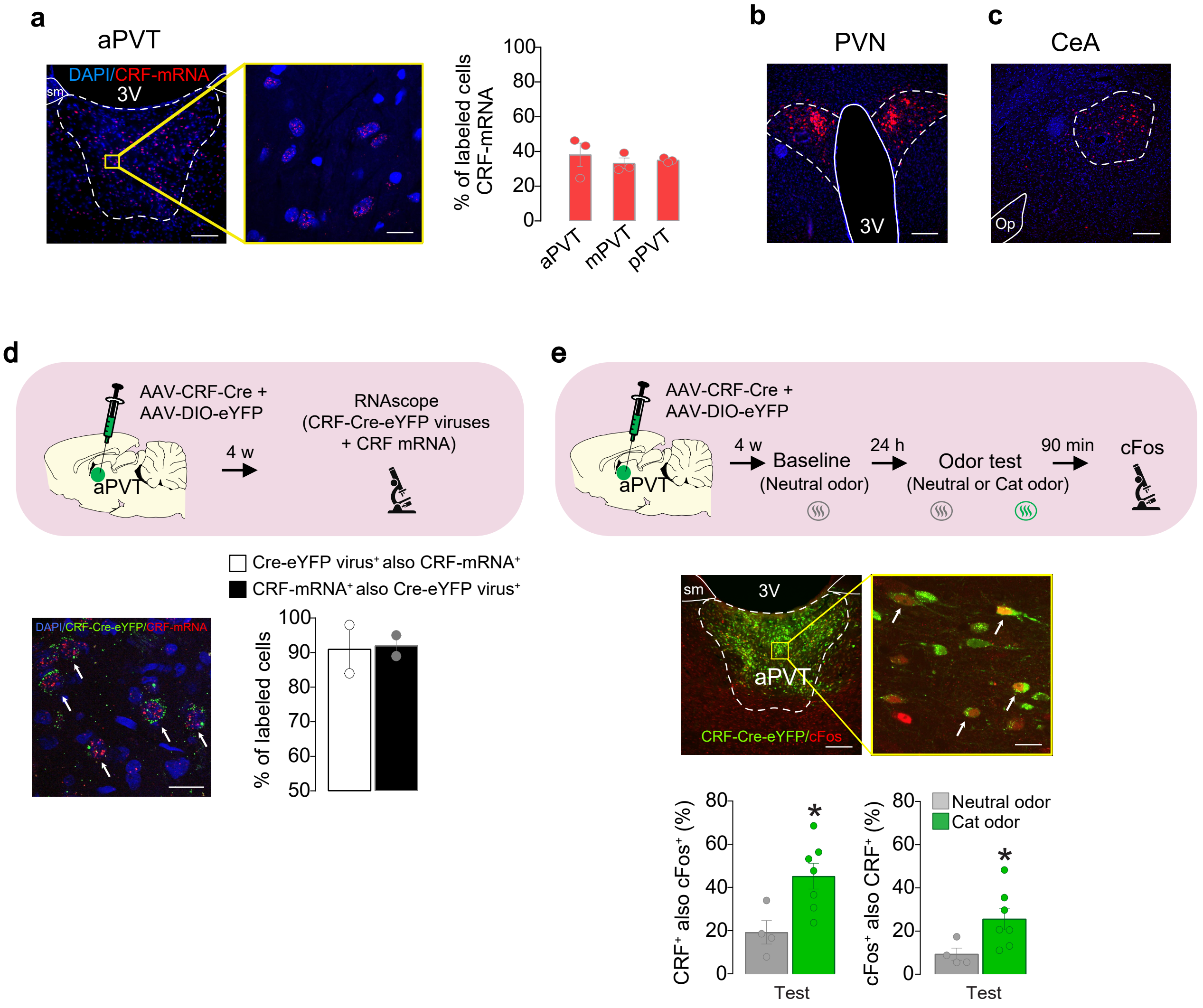

Supplementary Figure 7 (Related to Figure 5). Exposure to cat odor increases cFos expression in aPVT^{CRF} neurons.

(a) Distribution of aPVT^{CRF} neurons along the anterior-posterior axis of PVT identified by fluorescent *in situ* hybridization (RNAScope). Left: Representative micrograph of cells expressing CRF-mRNA in aPVT. Inset: High-magnification of the same micrograph. Right: Quantification of the total of CRF positive cells along the aPVT, medial PVT (mPVT) and posterior PVT (pPVT). No differences in the expression of CRF-mRNA were observed along the antero-posterior axis of PVT (Blue label, DAPI, red label, CRF-mRNA). **(b-c)** Representative micrographs of cells expressing CRF-mRNA in the (b) paraventricular nucleus of hypothalamus (PVN) and (c) central nucleus of the amygdala (CeA), two brain regions known for expressing high levels of CRF. This experiment was repeated in 3 rats with similar results. **(d)** Top, Timeline and schematic for the CRF-Cre-eYFP viral vectors validation. Rats injected with a mix of AAV-CRF-Cre + AAV-DIO-eYFP viruses into the aPVT were perfused 4 weeks later for RNAScope identification of CRF-mRNA. Bottom left, Representative micrograph of cells expressing AAV-CRF-Cre + AAV-DIO-eYFP (green label) that were colabeled for CRF-mRNA (red label, white arrows). Bottom right, Quantification of the total of cells expressing CRF-Cre + AAV-DIO-eYFP that were also labeled with CRF-mRNA (white bar), or cells labeled with CRF-mRNA that also expressed CRF-Cre + AAV-DIO-eYFP (black bar). **(e)** Cat odor exposure activated aPVT^{CRF} neurons. Top: Timeline of the cat odor-induced neuronal activity test. Naïve rats expressing eYFP under the control of AAV-CRF-Cre in aPVT were exposed to either neutral odor or cat odor for 10 min, and then perfused 90 min later for immunohistochemical analysis of cFos. Center: Representative micrograph showing aPVT^{CRF} neurons (green label) expressing immunoreactivity to cFos (red). Inset: High-magnification of the same micrograph. White arrows show examples of colabeled cells. Bottom left: Cat odor exposure (green bar, n = 7) significantly increased the percentage of CRF positive cells that were cFos positive, when compared to neutral odor controls (gray bar, n = 4; Unpaired Student's t test, P = 0.017, t = 2.89). Bottom right: Cat odor exposure also increased the percentage of cFos positive cells that were CRF, when compared to neutral odor controls (Unpaired Student's t test, P = 0.047, t = 2.30). Scale bars: 100 μm; Inset scale bars: 25 μm. All data shown as mean ± SEM.

Supplementary 8

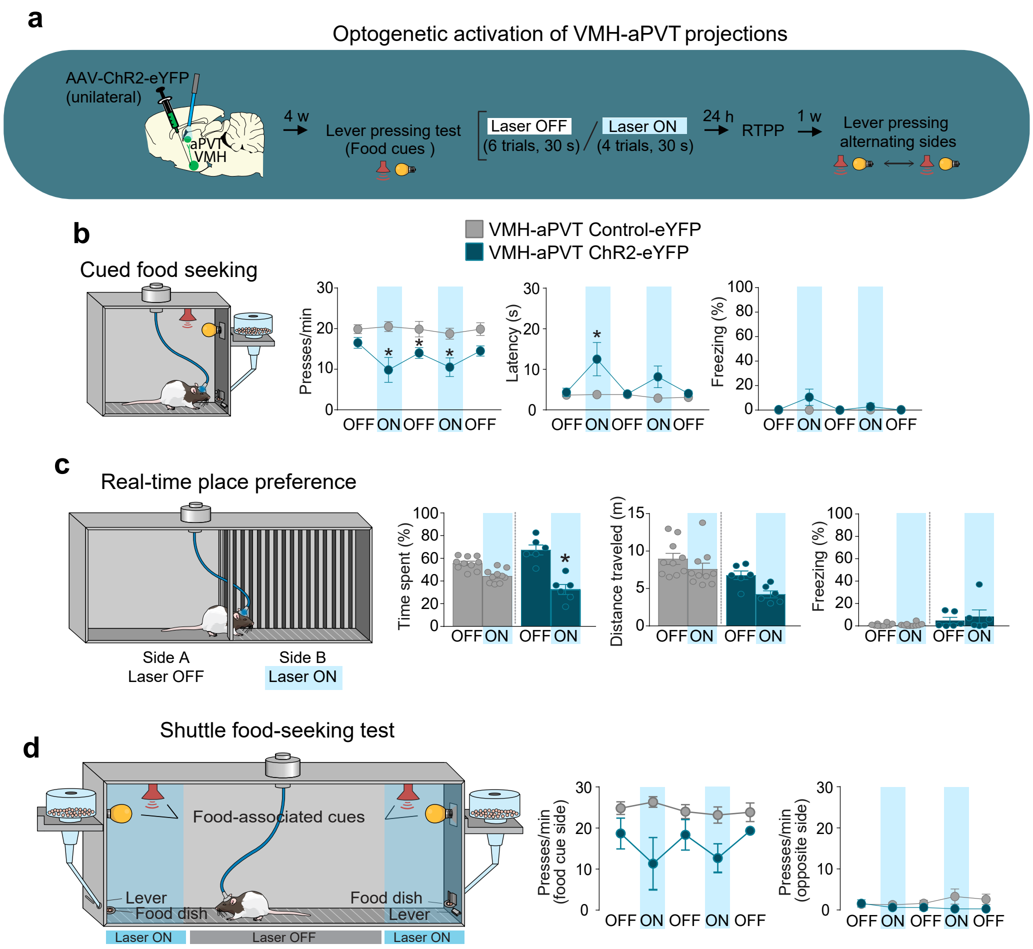

Supplementary Figure 8 (Related to Figure 9). Photoactivation of VMH-aPVT projections suppresses food seeking, induces avoidance behavior, and mimics the cat odor-induced conflict during a shuttle food-seeking test.

(a) Top, Timeline of the cued food seeking test during optogenetic activation of VMH-aPVT projections. **(b)** Left, Schematic of the cued food seeking test. Center, Optogenetic activation of VMH-aPVT projections (dark blue circles, n = 6) in a neutral context reduced the number of lever presses ($F_{(4, 48)}$ = 2.65, P = 0.044) and increased the latency to press the lever ($F_{(4, 48)}$ = 3.46, P = 0.014), when compared to eYFP controls (gray circles, n = 8). Right, No difference was found in the percentage of time rats spent freezing during the illumination ($F_{(1, 12)}$ = 2.74, P = 0.12). Blue shaded area represents laser-on trials (20 Hz, 20 ms pulse width,15 mW, 30 s duration). Each circle represents the average of two consecutive trials**. (c)** Left, Schematic of the real time place preference test. Center, photoactivation of VMH-aPVT projections (dark blue bars, n = 6) reduced both the percentage of time spent ($F_{(1, 14)}$ = 5.81, P = 0.031) in the side of the chamber paired with laser stimulation (Side B), when compared to eYFP controls (gray bars, n= 10). Blue shaded areas represent the sum of all laser-on epochs (20 Hz, 20 ms pulse width,20 mW). Right, No difference was found in the distance ($F_{(1,14)}$ = 2.15, P = 0.16) traveled and the percentage of time rats spent freezing during the illumination ($F_{(1,14)}$ = 0.44, P = 0.51). **(d)** Left, Schematic of the shuttle food-seeking test. Rats were trained to press for sucrose in the side of the chamber in which the audiovisual cue was presented (30 s). During the test session, the laser was activated each time the animals entered the food area in the presence of the audiovisual cue, and was kept on until either the animals leave the area or the offset of the cue. Photoactivation of VMH-aPVT projections at the onset of the food area entry reduced the number of lever presses during the food cue presentation without changing lever presses in the opposite side of the chamber. This experiment was repeated with similar results in 3 rats expressing ChR2 in VMH-aPVT neurons and 6 control rats expressing eYFP in the same cells. Two-way repeated-measure ANOVA followed by Bonferroni post hoc test. Data shown as mean ± SEM. *p < 0.05. See also **Supplementary Movie 10**.
