# Supplementary material for: A hypothalamic-thalamostriatal circuit that controls approach-avoidance conflict in rats
Source: Nat Commun. 2021 May 4;12:2517. doi: 10.1038/s41467-021-22730-y (PMC8097010; doi:10.1038/s41467-021-22730-y)
Supplement: Supplementary file 3 — Description of Additional Supplementary Files [file 41467_2021_22730_MOESM3_ESM.pdf]

Description of Additional Supplementary Files

File Name: Supplementary Movie 1
Description: **Representative video of the predator odorinduced defensive behavior and contextual fear.** The first part of the video shows rat's baseline activity in the presence of a neutral odor (distilled water) on day 1. The second part of the video shows rat's defensive behaviour in the presence of predator odor (cat saliva, located in a metal mesh in the left side of the chamber), with a 16x speed video showing that the rat exhibits risk-assessment behavior and stays in the hidden area during most of the session on day 2. The third part of the video shows the same rat expressing defensive behavior to the context (in the absence of cat odor) on day 3.

File Name: Supplementary Movie 2
Description: **Representative video of the predator odor vs. food-seeking conflict test. The** first part of the video shows the rat pressing a lever for food after the onset of the food-associated audiovisual cue in the presence of a neutral odor (distilled water). The second part of the video shows a suppression in lever pressing induced by the presence of the predator odor (cat saliva, located in a metal mesh in the left side of the chamber), as described in Figure 1.

File Name: Supplementary Movie 3
Description: **Representative video of the conflict test during chemogenetic inhibition of aPVT-NAc projections.** (Top) A control rat expressing mCherry in aPVT-NAc neurons shows robust defensive behaviors and suppressed food-seeking responses during the conflict test. (Bottom) A rat expressing hM4Di in aPVT-NAc neurons shows reduced defensive behaviors and restored food-seeking responses during the conflict test. Both animals were administered with CNO.

File Name: Supplementary Movie 4
Description: **Representative video of the conflict test during chemogenetic inhibition of $aPVT^{CRF}$ neurons.** (Top) A control rat expressing mCherry in $aPVT^{CRF}$ neurons shows robust defensive behaviors and suppressed food-seeking responses during the conflict test. (Bottom) A rat expressing hM4Di in $aPVT^{CRF}$ neurons shows reduced defensive behaviors and restored food-seeking responses during the conflict test. Both animals were administered with CNO.

File Name: Supplementary Movie 5
Description: **Representative video of the cued foodseeking test during photoactivation of $aPVT^{CRF}$ neurons.** (Left) A rat expressing ChR2 in $aPVT^{CRF}$ neurons shows regular lever pressing during the audiovisual cue presentation when the laser is off. (Right) The same rat shows a clear suppression in food-seeking behavior when the laser is on.

File Name: Supplementary Movie 6
Description: **Representative video of the real-time place preference test during photoactivation of $aPVT^{CRF}$ neurons.** (Top) A control rat expressing EYFP in $aPVT^{CRF}$ neurons explores equally both sides of the chamber when the left side was paired with laser illumination of $aPVT^{CRF}$ neurons. (Bottom) A rat expressing ChR2 in $aPVT^{CRF}$ neurons and implanted with optical fibers in the NAc shows avoidance responses to the left side of the chamber paired with laser illumination of $aPVT^{CRF}$ neurons. The 8x speed video shows the preference of the animals along the session.

File Name: Supplementary Movie 7

Description: **Representative video of the cued foodseeking test during photoactivation of $aPVT^{CRF}$-NAc projections.** (Left) A rat expressing ChR2 in $aPVT^{CRF}$ neurons and implanted with optical fibers in the NAc shows regular lever pressing during the audiovisual cue presentation when the laser is off. (Right) The same rat shows a clear suppression in food-seeking behavior when the laser is on

File Name: Supplementary Movie 8
Description: **Representative video of the real-time place preference test during photoactivation of $aPVT^{CRF}$-NAc projections**. (Top) A control rat expressing EYFP in $aPVT^{CRF}$ neurons and implanted with optical fibers in the NAc explores equally both sides of the chamber when the left side was paired with laser illumination of $aPVT^{CRF}$-NAc projections. (Bottom) A rat expressing ChR2 in $aPVT^{CRF}$ neurons and implanted with optical fibers in the NAc shows avoidance responses to the left side of the chamber paired with laser illumination of $aPVT^{CRF}$-NAc projections. The 8x speed video shows the preference of the animals along the session.

File Name: Supplementary Movie 9
Description: **Representative video of the conflict test during chemogenetic inhibition of VMH-aPVT projections**. (Top) A control rat expressing mCherry in VMH-aPVT neurons shows robust defensive behaviors and suppressed food-seeking responses during the conflict test. (Bottom) A rat expressing hM4Di in VMH-aPVT neurons shows reduced defensive behaviors but maintained food-seeking suppression during the conflict test. Both animals were administered with CNO.

File Name: Supplementary Movie 10
Description: **Representative video of the shuttle foodseeking test during photoactivation of VMH-aPVT projections.** During the shuttle food seeking test, the laser was activated to illuminate VMHaPVT projections each time the animals entered the food area in the presence of the audiovisual cues, as an attempt to mimic the predator odor. The first part of the video shows that both the control rat expressing EYFP in VMH neurons and implanted with an optical fiber in aPVT (Top) and the experimental rat expressing ChR2 in VMH neurons and implanted with an optical fiber in aPVT (Bottom) exhibit regular lever pressing during the cue presentation (right side of the arena) when the laser is off. The second part of the video shows that the experimental rat (Bottom) exhibit reduced foodseeking behavior (left side of the arena) and increased avoidance response when the VMH-aPVT projections were illuminated.
