# Supplementary material for: A hypothalamic-thalamostriatal circuit that controls approach-avoidance conflict in rats
Source: Nat Commun. 2021 May 4;12:2517. doi: 10.1038/s41467-021-22730-y (PMC8097010; doi:10.1038/s41467-021-22730-y)
Supplement: Supplementary file 5 — Reporting Summary [file 41467_2021_22730_MOESM5_ESM.pdf]

nature research

Corresponding author(s): Fabricio Do Monte

Last updated by author(s): Mar 3, 2021

Reporting Summary

Nature Research wishes to improve the reproducibility of the work that we publish. This form provides structure for consistency and transparency in reporting. For further information on Nature Research policies, see our Editorial Policies and the Editorial Policy Checklist.

Statistics

For all statistical analyses, confirm that the following items are present in the figure legend, table legend, main text, or Methods section.

n/a	Confirmed	
☐	☒	The exact sample size (*n*) for each experimental group/condition, given as a discrete number and unit of measurement
☐	☒	A statement on whether measurements were taken from distinct samples or whether the same sample was measured repeatedly
☐	☒	The statistical test(s) used AND whether they are one- or two-sided<br>*Only common tests should be described solely by name; describe more complex techniques in the Methods section.*
☒	☐	A description of all covariates tested
☐	☒	A description of any assumptions or corrections, such as tests of normality and adjustment for multiple comparisons
☐	☒	A full description of the statistical parameters including central tendency (e.g. means) or other basic estimates (e.g. regression coefficient) AND variation (e.g. standard deviation) or associated estimates of uncertainty (e.g. confidence intervals)
☐	☒	For null hypothesis testing, the test statistic (e.g. *F*, *t*, *r*) with confidence intervals, effect sizes, degrees of freedom and *P* value noted<br>*Give P values as exact values whenever suitable.*
☒	☐	For Bayesian analysis, information on the choice of priors and Markov chain Monte Carlo settings
☒	☐	For hierarchical and complex designs, identification of the appropriate level for tests and full reporting of outcomes
☐	☒	Estimates of effect sizes (e.g. Cohen's *d*, Pearson's *r*), indicating how they were calculated

Our web collection on statistics for biologists contains articles on many of the points above.

Software and code

Policy information about availability of computer code

Data collection	Behavioral data were measured using ANY-maze 6 (Stoeling). In vivo single-unit electrophysiology data were obtained using a 64-channels neuronal data acquisition system (Omniplex, Plexon) integrated with a high-resolution video-tracking system (Cineplex, Plexon). Ex vivo Electrophysiology Recordings were acquired using Clampex 10.3 (Molecular Devices). Images were taken using NIS Element Software 5.0 (Nikon).
Data analysis	Behavioral data, cell counting, and ex vivo recording data were analyzed using Prism 7 (GraphPad). In vivo recording data were processed with Offline Sorter V3 (Plexon) and analyzed using NeuroExplorer 4 or 5 (NEXT Technologies) and MATLAB scripts. Ex vivo recording data were processed using custom macros written in IGOR Pro 8.0 (Wavemetrics). Images were analyzed with NIS Element Software 5.0 (Nikon).

For manuscripts utilizing custom algorithms or software that are central to the research but not yet described in published literature, software must be made available to editors and reviewers. We strongly encourage code deposition in a community repository (e.g. GitHub). See the Nature Research guidelines for submitting code & software for further information.

Data

Policy information about availability of data

All manuscripts must include a data availability statement. This statement should provide the following information, where applicable:

- Accession codes, unique identifiers, or web links for publicly available datasets
- A list of figures that have associated raw data
- A description of any restrictions on data availability

All the data that support the findings presented in this study are available from the corresponding author on reasonable request.

Field-specific reporting

Please select the one below that is the best fit for your research. If you are not sure, read the appropriate sections before making your selection.

- [x] Life sciences
- [ ] Behavioural & social sciences
- [ ] Ecological, evolutionary & environmental sciences

For a reference copy of the document with all sections, see nature.com/documents/nr-reporting-summary-flat.pdf

Life sciences study design

All studies must disclose on these points even when the disclosure is negative.

Sample size	We used a sample size that is similar to previous published studies (e.g. Do Monte et al. 2015 Nature). For behavioral experiment, we used sample size of around 5-10 rats from 2-3 replications. For in vivo electrophysiology experiment, we used sample size greater than 20 neurons. For slice recording, we used sample size around 10 neurons from 2-3 rats. Similar number of animals were used in experimental and control groups.
Data exclusions	Animals without correct optical fiber or electrode placement and adequate viral expression were excluded from the study.
Replication	All data were obtained from at least two cohorts tested at different occasions in the presence of control animals. The results from each cohort were successfully replicated.
Randomization	All animals were randomly assigned to experimental or control groups and were tested in a counterbalanced order. For experiments involving cat odor presentation, control groups were examined in the first and last trials to make sure that the efficacy of the cat odor in inducing defensive responses was maintained during the whole session.
Blinding	Most behavioral data was generated using an automated video tracking software (Any-MAZE). The experimenter was blind to the experimental groups when doing manual scoring of behavioral data and manual counting of immunohistochemistry images.

Reporting for specific materials, systems and methods

We require information from authors about some types of materials, experimental systems and methods used in many studies. Here, indicate whether each material, system or method listed is relevant to your study. If you are not sure if a list item applies to your research, read the appropriate section before selecting a response.

Materials & experimental systems

n/a	Involved in the study	
[ ]	[x]	Antibodies
[x]	[ ]	Eukaryotic cell lines
[x]	[ ]	Palaeontology and archaeology
[ ]	[x]	Animals and other organisms
[x]	[ ]	Human research participants
[x]	[ ]	Clinical data
[x]	[ ]	Dual use research of concern

Methods

n/a	Involved in the study	
[x]	[ ]	ChIP-seq
[x]	[ ]	Flow cytometry
[x]	[ ]	MRI-based neuroimaging

Antibodies

Antibodies used	Primary antibody: Rabbit anti-cFos (EMD Millipore, ABE457), Rabbit anti-GFP (Invitrogen, A11122).<br>Secondary antibody: Goat anti-rabbit IgG antibody (H+L), biotinylated (Vectorlabs, BA-1000), Goat anti-rabbit IgG H&L (Alexa Fluor® 594, abcam, ab150080), Cy2 Donkey Anti-Rabbit IgG (H+L) (Jackson Immuno Research, 711-225-152).
Validation	Validation of Rabbit anti-cFos (EMD Millipore, ABE457) by manufacture: Immunohistochemistry (Paraffin) Analysis, A 1:1,000 dilution from a representative lot detected c-Fos in rat brain tissue sections (https://www.emdmillipore.com/US/en/product/Anti-c-Fos-Antibody,MM_NF-ABE457#anchor_Applications).<br>Validation of Rabbit anti-GFP (Invitrogen, A11122) by manufacture: test for Immunohistochemistry (IHC) in 1:200-1:2000 dilution in rat (https://www.thermofisher.com/antibody/product/GFP-Antibody-Polyclonal/A-11122)<br>These antibodies were tested by the manufacturer and have been widely used in the same species and application in previous studies. We have performed a pilot experiment in our lab to validate the antibody concentrations.

Animals and other organisms

Policy information about studies involving animals; ARRIVE guidelines recommended for reporting animal research

Laboratory animals	Male and female Long-Evans hooded adult rats (Charles Rivers Laboratories) with 3-5 months of age and weighing 330-450 g at the

	time of the experiment were used.
Wild animals	No wild animals were used.
Field-collected samples	No field-collected samples were used.
Ethics oversight	All experimental procedures were approved by the Center for Laboratory Animal Medicine and Care of The University of Texas Health Science Center at Houston. The National Institutes of Health guidelines for the care and use of laboratory animals were strictly followed in order to minimize any potential discomfort and suffering of the animals.

Note that full information on the approval of the study protocol must also be provided in the manuscript.
